# Foreign Summary

**Published:** 1839-02-02

**Authors:** 


### FOREIGN SUMMARY.

*Reduction of old Dislocations.*—Mr. Syme has lately reported in the Edinburgh Medical and Surgical Journal, two cases of reduction of a dislocation of the thigh-bone,—one on the dorsum ilii, of nine weeks' standing—the other into the ischiatic notch, of six weeks' standing. The subject of the first case was thirty-six years of age; and the reduction was effected in forty minutes, by the pulleys, after a bleeding, a warm bath, and the administration of four grains of tartar emetic. The subject of the second case was fifty-six years of age: a first attempt of three quarters of an hour's induration failed to effect the reduction; but a second, the following day, in a very short time, proved successful.

---

*Spontaneous partial Inversion of the Uterus.* By William Lawrence.—Sarah Smith, thirty-two years of age, a maid-servant, was admitted into the hospital on June 12th, 1838. She had always enjoyed good health, and felt herself quite well at the time of her admission. Three years ago she had borne a child, and the catamenia had been perfectly regular since that period. She had menstruated three weeks before she came to the hospital. She represented to me that she had a swelling in the private parts. I found, on examination, a tumour hanging from the external organs, as large as my fist. It was largest in the middle; a little smaller above and below. Observing a transverse fissure in the middle of its inferior end, I at first supposed the case to be a complete prolapsus uteri, but could not recognise the usual appearance of the os tincæ. The surface of the swelling, in its upper two-thirds, was smooth, pale, and nearly dry: this was obviously the vagina completely inverted. The lower third was a soft, almost villous, red surface, moistened by colourless mucus, and was soon recognised by the characteristic folds as the cervix uteri inverted. A defined line marked the boundary between the vaginal and uterine portions of the tumour. She stated that she had experienced a bearing down and uneasiness for five months; that a protrusion had occurred at the external parts for three months, going up of itself after she had lain down at night; that the swelling had been down permanently for the last three weeks, though she had continued to perform her duties as a domestic up to the very day of her coming to the hospital. The mucous membrane of the inverted cervix uteri was healthy; and the cavity of the uterus, into which I introduced the end of my finger, was perfectly so. I could not detect inflammation, enlargement, or any other morbid change. The exposed membrane had secreted abundantly, for the chemise was completely saturated and stiffened with an almost colourless discharge. My inquiries failed to elicit any circumstances from which the cause and mode of production of this unusual change could be explained. When the patient had gone to bed, I covered the protrusion with a soft cloth, and pressed it upwards with the hand. It was necessary to exert considerable force, under which it suddenly receded, the urine being forced out at the same moment. I introduced my fingers into the vagina, to ascertain that the uterus was restored to its natural state, and that the os tincæ was in its right place. A portion of sponge dipped in a solution of alum was then introduced into the vagina and kept there, the patient being confined to bed. She menstruated at the return of the regular period. She was kept in bed, and used the sponge for three weeks. She was then allowed to get up, still continuing to introduce the sponge. She was discharged quite well on July 25th. I saw her in the latter part of August, when she had again menstruated, and had experienced no return of the protrusion.—*Lon. Med. Gaz.*

---

*Sketch of Sir Charles M. Clarke.* By Dr. James Johnson.—Sir Charles was born in 1782, and, consequently, is in his fifty-sixth year. He was a fellow-student of our own, in Great Windmill street, under the late Mr. Wilson, and the present Mr. Thomas. Having served a short time in the army, he entered on practice under the auspices of his brother; and commenced lectures with him in 1804. Although a very different character from Sir Henry Halford, whom we have before sketched, yet a great deal of what we have said respecting the senior baronet will apply to the junior. But their professional avocations ran in very different channels. In an overwhelming majority of instances—in forty-nine cases out fifty, the *lying-in* chamber is anything but a *dying-in* apartment. In the former, the doctrines of Malthus and Martineau are thrown to the winds; and the addition of every unit, doublet, or even triplet to the sum total of the population, is hailed with rapture, and announced with glee, throughout the puerperal mansion. Never was man or midwife better calculated to enjoy the hilarity of the scene, or augment its intensity, than Sir Charles Mansfield Clarke! This amiable and talented physician seems as though he had been born for beguiling the tedious hours of labour—*taking* the pains from the fair sufferer—and by his looks and language inspiring hope, and banishing every shadow of despondency. To an inexhaustible flow of animal spirits, Sir Charles adds the auxiliaries of wit, humour, anecdote, and the most joyous of countenances. But one remarkable and highly useful talent he possesses in an extraordinary degree—the happy facility of explaining and elucidating those numerous subjects which naturally occupy attention, and start inquiries in a parturient bed-chamber. Woman is always a curious and inquisitive creature; but when the *chef-d'œuvre* of Nature's works—the evolution of man—is in progress, her curiosity, and that of her attendants, are excited to the utmost stretch. On these occasions, the ingenuity of Sir Charles is never at fault. A screw, a wedge, a nut-cracker, a smoke-jack, or any of the most common implements, or familiar agents in mechanics, art, or science, furnishes his fertile brain with prompt illustrations of the complex and wonderful machinery which is in operation for bringing into this world an ample supply of human beings to compensate the havoc produced by the scythe of Time, the sword of war, and the ravages of disease. These, however, are only extrinsic, though important accomplishments in the lying-in chamber. Sir Charles has resources at command of a much higher order, and a more intellectual character. He had the good fortune of having an elder brother of prime talents, and large experience, who ushered him into practice, assisted him by his counsel, and shielded him with his name. If such inestimable advantages are comparatively thrown away on some people, it was not so with the subject of this memoir. Sir Charles was born with brains in his head, which is more than every one can boast of—and he did not put his brains into his pocket, whatever else may have found its way there. By means of a quick perception, keen eye, and inexhaustible energy, knowledge rapidly accumulated, and its valuable products were as rapidly diffused over an extensive field of practice. The consequences were, a princely fortune and an unbounded reputation, before the age of fifty years! Envy, too, which follows wealth and fame, almost as regularly as the shadow follows the substance, has not pursued the personage in question,—or, if it did, it skulked in holes and corners unseen, unheard. Sir Charles has been liberal to his brethren, and they have been just towards him. Few other men could roll away to the coast or country, for five or six months annually—and return with a flowing sheet to a spring-tide of practice. Nothing but the unlimited confidence of the public, and the well-earned respect of the profession, would enable a medical man thus to sally alternately from the vortex of the metropolis to the "otium cum dignitate" of rural life, with undiminished influence or celebrity.—*London Medico-Chirurgical Review.*

